# CRISPR/Cas13-Based Anti-RNA Viral Approaches

**DOI:** 10.3390/genes16080875

**Published:** 2025-07-25

**Authors:** Xiaoying Tan, Juncong Li, Baolong Cui, Jingjing Wu, Karl Toischer, Gerd Hasenfuß, Xingbo Xu

**Affiliations:** 1 German Center for Cardiovascular Research (DZHK), Partner Site Göttingen, Robert-Koch-Str. 42a, 37075 Göttingen, Germany; xiaoying.tan@med.uni-goettingen.de (X.T.); juncong.li@med.uni-goettingen.de (J.L.); baolong.cui@med.uni-goettingen.de (B.C.); jingjingwu@usf.edu (J.W.); ktoischer@med.uni-goettingen.de (K.T.); hasenfus@med.uni-goettingen.de (G.H.); 2 Clinic for Cardiology and Pulmonology, University Medical Center Göttingen, Robert-Koch-Str. 40, 37075 Göttingen, Germany

**Keywords:** CRISPR/Cas13, RNA virus, SARS-CoV-2, COVID-19, crRNA, antiviral therapy

## Abstract

RNA viruses pose significant threats to global health, causing diseases such as COVID-19, HIV/AIDS, influenza, and dengue. These viruses are characterized by high mutation rates, rapid evolution, and the ability to evade traditional antiviral therapies, making effective treatment and prevention particularly challenging. In recent years, CRISPR/Cas13 has emerged as a promising antiviral tool due to its ability to specifically target and degrade viral RNA. Unlike conventional antiviral strategies, Cas13 functions at the RNA level, providing a broad-spectrum and programmable approach to combating RNA viruses. Its flexibility allows for rapid adaptation of guide RNAs to counteract emerging viral variants, making it particularly suitable for highly diverse viruses such as SARS-CoV-2 and HIV. This review discusses up-to-date applications of Cas13 in targeting a wide range of RNA viruses, including SARS-CoV-2, HIV, dengue, influenza, and other RNA viruses, focusing on its therapeutic potential. Preclinical studies have demonstrated Cas13’s efficacy in degrading viral RNA and inhibiting replication, with applications spanning prophylactic interventions to post-infection treatments. However, challenges such as collateral cleavage, inefficient delivery, potential immunogenicity, and the development of an appropriate ethical framework must be addressed before clinical translation. Future research should focus on optimizing crRNA design, improving delivery systems, and conducting rigorous preclinical evaluations to enhance specificity, safety, and therapeutic efficacy. With continued advancements, Cas13 holds great promise as a revolutionary antiviral strategy, offering novel solutions to combat some of the world’s most persistent viral threats.

## 1. Introduction

### 1.1. Overview of RNA Viruses

RNA viruses are a group of widespread pathogens responsible for infectious diseases in humans, animals, and plants [1,2,3]. A universal structural feature of RNA viruses is the presence of untranslated regions (UTRs) around one or more open reading frames (ORFs) at the 5′and 3′ ends, often containing sequences or conserved structures required for replication regulation [4,5]. Due to physical and structural limitations in RNA genome stability and the low fidelity of RNA polymerase, RNA virus genome sizes vary by an order of magnitude [6,7], with the larger genomes consisting of all class I dsDNA viruses [3]. Coronaviruses possess some of the largest positive-sense single-stranded genomes among RNA viruses, typically ranging from 27 to 32 kilobases. The SARS-CoV-2 genome, for example, is approximately 29.8 kb [8,9]. RNA viruses are distinguished by their ability to evolve rapidly, largely due to their reliance on RNA-dependent RNA polymerases (RdRPs) for replication. These polymerases lack proof reading mechanisms, resulting in frequent errors during replication [10]. RNA instability and high replication error rates allow RNA viruses to exhibit high genetic diversity and environmental adaptability, which enables them to rapidly adapt to new hosts and rapidly introduce genetic changes [11,12]. Alterations in viral genetic material drive phenotypic and population-level changes, including altered virulence, host tropism, antiviral resistance, and immune evasion, thereby facilitating novel pathogen emergence [13,14]. The continuous emergence of new strains and variants poses a major challenge to public health. This class of viruses includes those responsible for the most severe human health diseases, such as SARS-CoV-2 (the causative agent of COVID-19), HIV (which causes AIDS), and influenza viruses that cause seasonal influenza epidemics and global pandemics.

In addition to their impact on human health, RNA viruses can also affect animals. Porcine reproductive and respiratory syndrome virus (PRRSV) is a major pathogen in pigs [1], causing significant economic losses to the livestock industry. Among the various potential zoonotic pathogens, RNA viruses warrant particular attention due to their high mutation rates and cross-species transmissibility. For example, human infection with avian influenza viruses (AIVs) is an ongoing public health threat. The main risk factor for human infection with AIVs is direct exposure to infected poultry, particularly in regions such as Asia and Egypt, where live poultry markets are common [15]. It is very likely that a recent ancestor of the SARS-CoV-2 virus infected bats and possibly other intermediate species [16]. Notably, RNA viruses have emerged as significant zoonotic pathogens originating from wildlife. Recent studies have consistently identified RNA viruses as the predominant agents of emerging diseases in humans, accounting for approximately 44% of all emerging infectious diseases, with reported ranges between 25% and 44% across different studies. In contrast, bacteria contribute to 10–49% of these diseases, surpassing other parasitic groups such as fungi (7–9%), protozoa (11–25%), and helminths (3–6%). Therefore, understanding animal health dynamics and human–animal interactions is crucial for effectively addressing the challenges posed by these pathogens.

### 1.2. Current Antiviral Challenges

Antiviral vaccines and therapeutics constitute the primary strategies for the prevention and control of pandemic viral infections. In recent years, these vaccines and drugs have been extensively employed in the management and prevention of major infections caused by influenza viruses and SARS-CoV-2. Despite significant progress, major challenges remain. Influenza viruses often develop resistance to antivirals such as oseltamivir, while emerging SARS-CoV-2 variants—such as Delta and Omicron—have demonstrated reduced sensitivity to current vaccines. Likewise, resistance to antiretroviral therapy (ART) continues to complicate the long-term management of HIV. These issues underscore the significant challenges posed by RNA viruses, which are largely due to to their unique biological characteristics. This is because existing vaccines and drugs mainly target specific viral proteins, inducing limited immune responses. However, the structure and life cycle of viruses are often complex, the replication mechanism of some viruses is still unclear, and the high mutation rate of RNA viruses often alters viral proteins. Such mutations lead to the emergence of new variants, resulting in a progressive decline in the efficacy of existing vaccines and therapeutics, which must be continually updated to keep pace with evolving viral strains causing changes in viral proteins due to viral mutations and the emergence of potential new variants, resulting in a continuous decline in the protective and therapeutic effects of existing vaccines and drugs, which need to be continuously updated to cope with new viral variants.

### 1.3. Emergence of Cas13

Clustered regularly interspaced short palindromic repeats (CRISPR) were initially identified as part of an adaptive immune system in prokaryotes, such as bacteria and archaea, which provides defense against viral infections. The term “short palindromic repeats” refers to repetitive DNA sequences found within prokaryotic genomes. The CRISPR system was first discovered in *Escherichia coli* by the Japanese scientist Yoshizumi Ishino and his team in 1987, although its functional role was not understood at the time [17]. In 2002, a team led by Ruud Jansen identified and formally named CRISPR/associated proteins (Cas) [18]. A typical CRISPR/Cas system is composed of Cas genes, leader sequences, repeats, spacers, and tracrRNA, each playing a crucial role in the mechanism of adaptive immunity.

The CRISPR/Cas system is characterized by Cas proteins that are organized into two primary classes, each further divided into three subtypes. The first class comprises multi-protein effector complexes, which consist of several distinct Cas proteins, each tasked with specific functions. This class encompasses types I, III, and IV. In contrast, the second class consists of single-protein effector complexes, which are capable of performing their functions through a single Cas protein. This class includes type II (Cas9), type V (Cas12), and type VI (Cas13) (Table 1). Significantly, the second class of systems is distinguished by its simplicity, efficiency, and ease of use, making it the leading choice for gene editing applications [19,20,21]. Cas9, in particular, is the most widely recognized member of this class, known for its ability to target DNA for precise editing. In 2013, it was first applied to gene editing in mammalian cells [20], and in 2020, gene editing therapy was first performed directly in a human body. In the same year, the Nobel Prize in Chemistry was awarded to Emmanuelle Charpentier and Jennifer Doudna for their outstanding contributions to CRISPR/Cas9 research [22]. However, Feng Zhang’s team was the first to identify the distinctive capability of the Cas13 protein family to specifically target and cleave RNA rather than DNA [23,24] (Table 1). Building on this unique feature, they developed a nucleic acid molecular diagnostic technology utilizing the Cas13 system [25]. Over the past decade, the CRISPR/Cas system has distinguished itself among various gene editing technologies, demonstrating significant potential for application in viral genome editing. It is anticipated to become a novel therapeutic approach for treating viral infectious diseases. Cas13, in particular, recognizes specific RNA sequences via CRISPR RNA (crRNA) and cleaves them with high specificity. This property makes Cas13 particularly suitable for targeting RNA viruses, whose genomes are composed entirely of RNA. The programmability of Cas13 allows for rapid re-design of crRNAs to target newly emerging viral strains, offering a flexible solution to the challenge of viral mutations. This review explores Cas13’s potential as an antiviral tool, focusing on its mechanism of action, applications in RNA viral research, and the key challenges it faces before clinical implementation.

## 2. Cas13: Mechanism and Potential in Targeting RNA Viruses

### 2.1. Cas13 Discovery and Classification

Cas13 belongs to the Class 2, Type VI CRISPR system, and it is characterized by a single protein that performs both RNA recognition and cleavage, making it a unique and versatile tool for RNA manipulation. Cas13 was first discovered in 2015 as part of the expanding CRISPR family of proteins [24]. Feng Zhang’s team employed computational biology methodologies to identify the Cas13a system (also referred to as C2c2) within microbial metagenomic databases [23,24]. Their findings, which indicated that the presence of Cas1 and Cas2 genes in the CRISPR/Cas loci is not essential, inspired the development of a novel computational approach. This advancement led to the discovery of the Cas13b system, classified as the type VI-B system in 2017 [26,27]. By continuously refining these computational methods, Zhang’s team expanded their investigation to include smaller effectors in 2018, resulting in the discovery of the Cas13d system [28]. At approximately 930 amino acids in length, Cas13d is the smallest type VI CRISPR/Cas effector identified to date and is the most widely utilized subtype of Cas13. In 2021, Yang Hui’s research group introduced the Cas13X system, the smallest known RNA editing tool, consisting of only 775 amino acids, thereby enhancing its suitability for in vivo delivery [29]. Building on this, Yang’s team developed a high-fidelity Cas13X protein variant, hfCas13X, which exhibits high gene editing activity with exceptionally low off-target effects, demonstrating significant potential for applications in RNA editing-based in vivo gene therapy [30]. Each Cas13 isoform has different properties and is suitable for different applications. Cas13’s specificity is determined by its crRNA, a small RNA sequence complementary to the target viral RNA. Upon recognition of the target RNA, Cas13 undergoes a conformational change that activates its HEPN (Higher Eukaryotes and Prokaryotes Nucleotide-binding) domains, which are responsible for RNA cleavage. This activation results in precise cutting of the viral RNA, effectively halting viral replication. The features of the Cas13 members identified so far are summarized (Table 2).

### 2.2. Mechanism of Action

Cas13’s mechanism of action involves a three-step process that enables it to target and degrade viral RNA. The first step, recognition and binding, is facilitated by crRNAs that are complementary to specific regions of the viral RNA genome. These crRNAs guide the Cas13 protein to bind precisely to its target within the viral RNA. Upon successful binding, Cas13 undergoes a conformational change, activating its HEPN domains. These domains are crucial for the second step, as they cleave the target RNA at specific sites, effectively silencing the viral genome and preventing it from translating into viral proteins. The third step, collateral cleavage, occurs once Cas13 is activated. In this phase, the protein indiscriminately cleaves nearby RNAs in addition to the target. The collateral activity of Cas13 is inherent to its structural mechanism. Upon crRNA binding to the target RNA, the HEPN1 and HEPN2 domains within the NUC lobe are activated, forming a complete catalytic center located on the protein surface. This center engages substrates primarily through electrostatic interactions with the RNA phosphate backbone, without recognizing specific base sequences. Unlike Cas9 or Cas12, Cas13 lacks a PAM- or PFS-like gating mechanism, resulting in limited discrimination between target RNA and bystander RNAs [31]. Current engineering strategies focus on attenuating the HEPN domain interface to reduce collateral cleavage while preserving target specificity and efficiency.

While collateral cleavage can be advantageous in bacterial systems by ensuring comprehensive degradation of viral RNA, it poses a potential challenge in human therapeutic applications, as it risks degrading essential host RNAs and causing unintended side effects. Addressing this issue is critical for safely harnessing Cas13’s antiviral potential. Current strategies are to weaken the HEPN contact surface to reduce collateral activity and maintain a high targeting efficiency. RfxCas13d is the most widely used variant of Cas13. It can maintain high knockout efficiency as much as possible while maintaining high specificity and low collateral cleavage activity in mammalian cells [2,3]. It has been delivered in vivo to mice and used in the regulation of liver metabolism and nervous system [4,5,6,7]. However, the researchers found that the higher the endogenous gene expression level or the more RfxCas13d content, the stronger the off-target effect. Based on this, yang’s group developed hfCas13d with less potent off-target effects and a knockout efficiency that was less than 5% different from that of RfxCas13d. In addition, hfCas13X is a small-volume Cas13 variant that is more favorable for in vivo AAV delivery. Although the knockout efficiency of hfCas13X was lower than that of hfCas13d, there was almost no off-target effect [8].

### 2.3. Advantages of Cas13 over Traditional Antiviral Strategies

**Figure 1:**
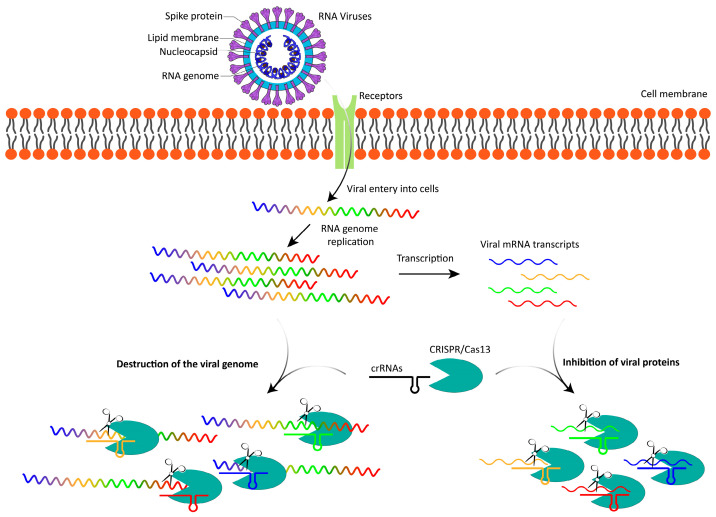
Cas13-mediated inhibition of viral replication. Upon the entry of an RNA virus into a host cell, the viral RNA genome is released into the cytoplasm, where it undergoes replication and is simultaneously transcribed into viral mRNA transcripts. Cas13 enzymes, guided by specific crRNAs, inhibit viral replication by directly targeting and degrading the viral RNA genome. Additionally, Cas13 can degrade viral mRNA transcripts, preventing the translation of viral proteins. By disrupting both viral genome integrity and protein production, Cas13 effectively blocks viral proliferation and reduces the expression of viral proteins. The colored regions of the RNA virus genome represent different coding sequences for viral proteins.

The Cas13 system offers several notable advantages over traditional antiviral approaches, positioning it as a promising tool in combating RNA viruses (Figure 1). One of its key strengths lies in its high specificity, as Cas13 can be programmed with crRNAs to precisely target and degrade RNA sequences unique to specific viruses. This precision minimizes off-target effects, making it well-suited for therapies requiring accurate viral RNA targeting. Additionally, Cas13 exhibits wide-ranging potential due to its versatility; it can be rapidly reprogrammed to address a wide range of RNA viruses. This capability enables swift development of new crRNAs to counter emerging strains, offering a significant advantage over traditional antivirals that often require years to develop. Furthermore, Cas13’s programmability makes it adaptable for addressing rapidly evolving viral threats, including rapidly mutating viruses like novel variants of SARS-CoV-2, underscoring its flexibility and responsiveness in the fight against infectious diseases.

## 3. Applications of Cas13 in Targeting RNA Viruses

Viral nucleic acids serve as the primary targets for both virus detection and the development of antiviral therapeutics. Various gene editing technologies have the capability to specifically target and modify nucleic acid sequences, thereby facilitating viral nucleic acid detection and the creation of antiviral drugs. The Cas13 system stands out due to its remarkable sensitivity, efficiency, and safety. In recent years, the CRISPR/Cas system has found extensive application in virus detection, and there has been significant exploration into its potential for developing antiviral vaccines and drugs. This section primarily addresses the utilization of Cas13 in treatment as well as the detection of a range of human as well as non-human pathogenic viruses (treatment summarized in Table 3), with the aim of providing a comprehensive overview of the current advancements and serving as a reference for future research.

Cas13-mediated knockdown efficiencies reported across studies vary substantially, ranging from approximately 72% to over 99%. This variability arises from multiple factors, including the choice of Cas13 ortholog, crRNA design, target site accessibility, and delivery method (e.g., plasmid transfection vs. RNP delivery). Differences in cellular context—such as target RNA abundance—and variability in readouts, including transcript levels, protein expression, or phenotypic outcomes, further contribute to these discrepancies. Reproducibility remains a central challenge in the field, exacerbated by the lack of standardized crRNA design algorithms, inconsistent evaluation criteria for knockdown, and divergent experimental platforms (using different Cas13 orthologs) across laboratories. Addressing these issues will require the development of standardized protocols, benchmarking datasets, and systematic cross-platform comparisons to improve reproducibility and enable robust assessment of Cas13-based technologies.

### 3.1. SARS-CoV-2

The global COVID-19 pandemic has driven significant research into Cas13’s potential to combat SARS-CoV-2. Abbott’s team has successfully developed a Cas13-based strategy known as PAC-MAN (Prophylactic Antiviral CRISPR in Human Cells). This approach specifically targets highly conserved regions of the SARS-CoV-2 genome, facilitating the degradation of SARS-CoV-2 RNA within a human lung epithelial cell model [32]. Blanchard tested Cas13a in a hamster model, where Cas13a was delivered via a nasal nebulizer. Treated hamsters showed significantly reduced viral loads in the lungs and less severe symptoms, illustrating the potential for Cas13-based treatments in respiratory infections like COVID-19 [34]. Zeng’s team used Cas13d to target multiple strains of SARS-CoV-2, including the Alpha, Beta, and Omicron variants, which have shown significant resistance to vaccines and antiviral drugs. In Vero E6 cells, Cas13d reduced viral replication by over 95%, demonstrating its efficacy in neutralizing the virus [33]. Considering the RNA genome of SARS-CoV-2 and the emergence of new variants, frequent mutations in the virus may lead to recognition failures by Cas13 systems. To address this, Wang’s group employed a comprehensive suite of bioinformatics techniques, including sequence alignment, structural comparison, and molecular docking, to identify conserved regions within the SARS-CoV-2 genome that are targetable by Cas13 [35]. Furthermore, Fareh et al. developed a Cas13b-based system specifically designed to inhibit the replication of various SARS-CoV-2 variants, including the variant of interest B.1.1.7, in infected mammalian cells [51]. Another study by Liu et al. used reprogrammed Cas13d effectors targeting NSP13, NSP14, and nucleocapsid transcripts of the SARS-CoV-2 genome and achieved >99% silencing efficiency in human cells including four different variants such as B.1, B.1.1.7 (Alpha), D614G B.1.351 (Beta), and B.1.617 (Delta). Based on bioinformatics data predictions, the designed crRNAs could almost 100% target the Omicron variant as well [38].

The inhibition efficiency can vary significantly and is largely dependent on the crRNA target region within the viral genome. Yu utilized mRNA-encoded Cas13b to target the pseudoknot site upstream of ORF1b. This approach was tested in Vero E6 cells and hACE2 transgenic mice. The results showed a 99% reduction in spike protein expression and a marked attenuation of viral replication [36]. Cas13 offers a promising approach to inhibit viral replication by targeting conserved regions of the viral genome.

Early diagnosis of viral infections and timely antiviral treatment are crucial in minimizing the risk of disease transmission. Nucleic acid testing is considered the gold standard for confirming viral infections, with fluorescent quantitative PCR being the most widely employed method for viral nucleic acid detection [52]. However, nucleic acid testing necessitates stringent requirements for sample preservation, nucleic acid extraction conditions, operator proficiency, and the operating environment. These demands pose challenges to large-scale early screening and diagnosis efforts [53]. In contrast, large-scale screening often relies on antigen detection methods based on colloidal gold, which are quick and easy to perform but suffer from low sensitivity and a tendency for false-negative results. Consequently, the development of a more efficient and user-friendly CRISPR-based platform presents a promising alternative for viral detection. Utilizing SHERLOCK technology for SARS-CoV-2 detection allows for the identification of novel coronavirus strains and their variants within one hour [54], with a minimum detection threshold of 10 to 100 copies/μL and a 97% concordance rate with clinical positive samples. This method significantly enhances sensitivity and specificity compared to traditional detection technologies. At the same time, Wilson’s group has designed a method called CREST (Cas13-Based, Rugged, Equitable, Scalable Testing) to further reduce the cost of using Cas13 for large-scale testing [55], and Arizti-Sanz’s team also innovatively integrated the Cas13 system, fluorescence monitoring, and smartphone applications into the SHINE detection system, achieving effective nucleic acid detection of SARS-CoV-2 [56]. It is anticipated that with ongoing optimization, the Cas13 system will evolve into a novel nucleic acid diagnostic tool.

### 3.2. HIV

According to data from the World Health Organization (WHO), by the end of 2022, approximately 39 million individuals globally were living with human immunodeficiency virus (HIV) [57]. Highly active antiretroviral therapy remains the primary treatment strategy for patients with HIV-1 [58]. However, HIV presents significant challenges due to its ability to integrate into the host genome and establish latent reservoirs that are resistant to current antiretroviral therapies [59]. Highly active antiretroviral therapy (HAART) is ineffective in eradicating these latent viral reservoirs, rendering HIV a chronic, incurable condition. The CRISPR/Cas system offers a novel approach for HIV treatment by targeting the HIV-1 genome to reduce infection or eliminate the virus [60]. In 2013, the CRISPR/Cas9 system was first utilized for HIV treatment. The Ebina team designed crRNAs to target specific HIV sequences in integrated proviral DNA and demonstrated that CRISPR/Cas9 could successfully inhibit HIV-1 genome expression, disrupt viral gene expression, and reduce viral replication in Jurkat cell lines, showing that targeting multiple sites in the genome simultaneously could enhance excision efficiency [61]. Excision Biotherapeutics initiated a Phase 1 clinical trial (NCT05144386) in 2022 to evaluate EBT-101, an in vivo Cas9-based therapy designed to excise integrated HIV proviral DNA from infected cells [62]. The approach employs intravenous AAV9-mediated delivery of Cas9 and dual crRNAs targeting conserved regions of the viral genome. Initial dosing was completed in a small cohort, with the trial meeting safety and tolerability endpoints. As of December 2024, EBT-101 completed a Phase 1/2a clinical trial. Interim results presented at the 27th ASGCT meeting in May 2025 showed that EBT-101 did not prevent viral rebound after ART interruption in three treated participants, highlighting the difficulty of translating preclinical success into clinical efficacy. One possible explanation is the insufficient delivery of the AAV9 vector. To address this, the company is actively optimizing the current AAV system and developing next-generation vectors with improved activity and manufacturability [62].

Combining CRISPR/Cas9 with classical ART, including fusion inhibitors, nucleoside reverse transcriptase inhibitors (NRTIs), non-nucleoside reverse transcriptase inhibitors (NNRTIs), integrase strand transfer inhibitors (INSTIs), and protease inhibitors (PIs), represents a synergistic strategy that can enhance antiviral efficacy, raise the barrier to resistance, and reduce drug doses and toxicity [63]. Such combinatorial approaches, which target both viral and host factors, may prove especially effective for durable suppression of HIV and broader RNA virus infections [63].

Unlike Cas9, Cas13 does not alter the DNA sequence, potentially allowing cells to tolerate and recover from off-target effects without permanent DNA damage [23,25]. The Cas13 system offers a promising approach by targeting HIV RNA in both active and latent infections. Yin showed that Cas13a could substantially reduce HIV RNA levels in HEK293T cells [45]. By targeting viral RNA, Cas13a effectively suppressed viral replication, highlighting its potential to diminish latent viral reservoirs, which remain a major obstacle in HIV cure research. The ability of Cas13 to specifically target RNA offers the possibility of disrupting the transcription of latent HIV, providing hope for reducing or even eradicating these viral reservoirs. This approach could lead to functional cures, effectively eliminating HIV from the body and reducing the dependence on lifelong antiretroviral therapy.

More recent advancements in Cas13-based HIV research further explored its potential in therapy and viral detection. In 2021, RfxCas13d (CasRx) combined with HIV-specific crRNAs effectively inhibited HIV-1 replication in cell models, primary CD4^+^ T cells, and reactivated latent HIV, demonstrating its therapeutic potential [46]. Later, a membrane-based digital Cas13a system enabled absolute quantification of HIV-1 viral particles without amplification, optimizing crRNA design for enhanced detection sensitivity [64]. In 2023, a point-of-care Cas13a-based method provided a stable, simple, and highly sensitive tool for early HIV diagnosis and treatment monitoring, reinforcing Cas13’s role in next-generation HIV management [65].

### 3.3. Dengue, Influenza, and Other RNA Viruses

Dengue virus and other flaviviruses, such as Zika and West Nile virus, are responsible for millions of infections annually, particularly in tropical regions. Cas13 has shown potential in inhibiting these viruses by directly targeting their RNA genomes. Li’s group applied Cas13a to target the NS3 gene of the dengue virus, which is crucial for viral replication [40]. By introducing Cas13a into infected Vero cells, they observed a 95% reduction in viral RNA levels within two days, demonstrating Cas13’s effectiveness in disrupting dengue virus replication. Santangelo’s research team was the first to report the use of lipid nanoparticles (LNPs) for delivering mRNA encoding Cas13a and crRNA targeting the dengue virus (DENV) type 2 and 3 genomes. This treatment was administered one day after infection with a lethal viral dose. The approach successfully reduced DENV RNA levels in a mouse model and protected the mice from mortality [42]. The team is now collaborating with TFF Pharmaceuticals to explore the feasibility of producing dry powder inhalation formulations, advancing the potential for practical and efficient therapeutic applications.

The team led by Chiara Zurla and Philip J. Santangelo at Emory University pioneered a method for delivering mRNA encoding Cas13a and a replicase targeting the highly conserved regions of the influenza virus PB1/PB2 and SARS-CoV-2 via PBAE polymers in 2021 [34]. This strategy utilizes CRISPR RNA (crRNA) specific to the nucleocapsid gene, effectively degrading viral RNA in lung tissue following aerosol inhalation. This approach has demonstrated efficacy in mitigating symptoms of viral infections and offers a therapeutic avenue for influenza A and SARS-CoV-2. Building on this, the researchers employed PBAE polymers to encapsulate the mRNA for delivery through a human aerosol inhalation device. Notably, even when administered 24 h post infection in mice, the viral RNA in lung tissue, as quantified by qPCR, was reduced by 90% over three days. Moreover, cytopathic effect (CPE) indicators were significantly diminished, underscoring the effectiveness of this strategy in inhibiting viral replication and providing therapeutic benefits post infection. mRNA-encoded LbuCas13a, along with two crRNAs targeting H1N1 and H3N2 strains, was tested in A549 cells and hamsters. Recently, another group developed a similar strategy utilizing Cas13 to inhibit influenza viruses [44]. They employed mRNA-encoded LbuCas13a combined with two crRNAs designed to target H1N1 and H3N2 strains. This approach was tested in both A549 cells and hamsters. In vitro studies demonstrated effective RNA degradation when the treatment was administered 24 h post infection. Furthermore, in vivo experiments in hamsters revealed a significant 1–2 log reduction in viral titers, underscoring the potential of this method for combating influenza infections. In a subsequent development in 2024, the highly mutagenic nature of influenza A virus (IAV) makes it a difficult target for vaccines and antivirals. Cas13 offers an alternative by targeting the RNA of various strains of influenza, potentially preventing viral replication before new mutations can arise. In cell culture models, Cas13a was shown to reduce IAV replication by fourfold, offering a potential therapeutic pathway for seasonal and pandemic influenza [43].

Similarly, Zika virus is a single-stranded RNA (ssRNA) virus. Hao Pei’s team successfully used Cas13 to cleave Zika virus ssRNA in mammalian cells and screened several effective targets on the Zika virus genome, such as gE-2, GNS1-1, GNS1-2, GNS3-1, and gNS4B-1. These targets were designed for the highly conserved regions in the Zika virus genome. Multiple Zika virus mutants can be targeted, thereby preventing the escape of the virus mutants [66].

Hepatitis C Virus (HCV), another RNA virus, has also been targeted with Cas13, particularly at its internal ribosome entry site (IRES). Early studies have demonstrated that Cas13 can significantly reduce HCV replication, paving the way for its use in treating chronic infections like hepatitis C [48]. Additionally, Cas13a-based diagnostic technology has demonstrated high sensitivity and specificity (81.9% as compared to 66.7% for conventional qPCR detection method) in detecting the Hepatitis delta virus (HDV). This technology shows promise as a potential tool for monitoring HDV infection progression and evaluating therapeutic efficacy [67].

Borna disease virus (BoDV-1) also contains an RNA genome, which infects the central nervous system of various animals, including humans, causing fatal encephalitis and persistent neuronal infections with neurobehavioral effects. Sasaki et al. showed that the Cas13 system effectively reduces viral mRNAs and genomic RNA in persistently infected cells, demonstrating its potential to suppress BoDV-1 in both acute and persistent infections [50].

## 4. Challenges and Limitations of Cas13-Based Antiviral Therapies

### 4.1. Collateral Cleavage and Off-Target Effects

While Cas13’s specificity is a key advantage, its collateral cleavage activity poses a significant challenge. Once activated, Cas13 can degrade other non-target RNAs in the vicinity, raising the risk of unintended degradation of host transcripts and cell toxicity. In human cells, such off-target RNA degradation could disrupt essential cellular functions, leading to cytotoxicity or unintended side effects. Another way to increase the cleavage specificity requires strict design of crRNAs.

### 4.2. Delivery Challenges

Efficiently delivering Cas13 and its associated crRNAs into target cells remains a major obstacle. Viral vectors, such as adeno-associated viruses (AAVs), are commonly used to deliver CRISPR systems but face limitations, including immune responses and limited capacity to deliver large cargo. Alternatively, non-viral delivery systems, such as LNPs, have shown promise, particularly for delivering mRNA-based therapeutics like Cas13, but achieving tissue-specific targeting remains a key challenge. As this review is specifically focused on Cas13-based antiviral strategies targeting RNA viruses, we have emphasized recent and application-oriented developments in this area. For readers seeking more detailed insights into CRISPR/Cas gene delivery strategies—including lipid nanoparticles, viral vectors, and conjugated delivery systems—we refer to several comprehensive reviews that offer in-depth analyses of these delivery platforms and their tissue-specific targeting approaches [68,69].

### 4.3. Immunogenicity

Another limitation of Cas13-based therapies is the potential for immunogenicity. Cas13 proteins, especially when delivered via viral vectors, may be recognized by the host immune system, triggering an immune response that reduces the effectiveness of the treatment. This is particularly concerning for chronic infections that require repeated administrations, as the immune system may neutralize the Cas13 proteins or delivery vectors over time.

### 4.4. Limited In Vivo Data

Although Cas13 has shown great promise in in vitro studies and some in vivo animal models, there is still limited data on its long-term safety and efficacy in humans. Most Cas13 antiviral studies have been conducted in cell lines rather than in live animal models. Few studies have tested Cas13 in mice or non-human primates, making it difficult to assess immune response, pharmacokinetics, and long-term safety. The complexity of viral infections in living organisms, such as tissue tropism and immune evasion, requires rigorous in vivo validation. Until now, Cas13 antiviral therapies have not yet entered advanced preclinical or clinical trials, limiting real-world validation.

### 4.5. Regulatory and Clinical Approval Hurdles

While Cas13-based antiviral therapies hold great potential, their clinical translation involves distinct regulatory pathways depending on their intended use—therapeutic or diagnostic. For therapeutic applications, regulatory approval typically requires extensive preclinical and clinical validation. Key steps include the following: 1. delivery optimization, demonstrating safe, stable, and tissue-specific delivery in relevant animal models; 2. preclinical safety studies, including assessments of toxicity, immunogenicity, and off-target effects; 3. clinical trials (Phases I–III) to establish safety, optimal dosing, efficacy, and long-term outcomes in humans. These steps must be conducted in compliance with international regulatory guidelines, such as those from the Food and Drug Administration (FDA), European Medicines Agency (EMA), and International Council for Harmonisation of Technical Requirements for Registration of Pharmaceuticals for Human Use (ICH), and aligned with ethical standards for gene editing technologies. At the time of this writing, two ongoing Phase 1 clinical trials are evaluating HG202, a one-time CRISPR/Cas13 RNA-editing therapy delivered via a single AAV vector, for the treatment of neovascular age-related macular degeneration (nAMD). HG202 targets VEGFA to inhibit choroidal neovascularization (CNV), aiming to reduce the need for frequent anti-VEGF injections and offer a therapeutic option for patients with poor or no response to existing therapies [70,71]. This gene editing approach may address both the efficacy and safety limitations of current long-term anti-VEGF treatments.

In contrast, diagnostic applications of Cas13—such as SHERLOCK-based platforms—follow a different regulatory route, typically overseen by agencies such as the FDA’s Center for Devices and Radiological Health (CDRH). These products may qualify for expedited review pathways such as Emergency Use Authorization (EUA), depending on the intended use and risk classification. For diagnostics, key regulatory considerations include analytical validity, sensitivity and specificity, and robustness in clinical settings.

By distinguishing between these regulatory frameworks, it becomes clear that the clinical development and approval of Cas13-based technologies will depend heavily on their application context, with therapeutics facing more stringent and prolonged evaluation compared to diagnostics.

### 4.6. Economic Considerations and Scalability

The cost-effectiveness and scalability of Cas13-based antiviral therapies are critical factors for their potential global health impact, particularly in low- and middle-income countries. While the programmability and modular nature of Cas13 offer advantages in terms of rapid adaptation to emerging RNA viruses, the production and delivery of these therapies—especially when relying on viral vectors or complex lipid nanoparticles—remain expensive and technically demanding. Furthermore, large-scale manufacturing of high-quality crRNAs and delivery components requires robust infrastructure and regulatory oversight. However, ongoing advances in RNA synthesis technologies, non-viral delivery systems, and point-of-care formulations may significantly reduce production costs and improve accessibility. To ensure equitable distribution and practical deployment of Cas13-based therapeutics, future efforts should prioritize scalable delivery platforms, cost-effective manufacturing strategies, and integration with existing healthcare systems.

## 5. Future Directions and Ethical Considerations

### 5.1. Research Advancements

Advancements in Cas13-based therapies are being driven by ongoing efforts to enhance specificity, optimize delivery mechanisms, and expand antiviral applications. Research is actively refining crRNA design and employing protein engineering to improve Cas13 specificity, aiming to reduce off-target effects and minimize collateral cleavage while preserving high on-target efficiency. Beyond traditional considerations for selecting the optimal target region and refining crRNA length and composition, significant advancements in computational tools for off-target prediction and crRNA selection are essential. Perturb-Seq, which integrates pooled Cas13 crRNA screening with scRNA-seq readouts, offers a powerful approach to generating large-scale data. This wealth of information can foster collaboration with bioinformaticians, enabling the development of machine-learning models to refine crRNA design. Ultimately, these efforts could lead to a universal computational tool capable of precisely selecting crRNAs for targeting RNA-based viruses with high specificity and efficiency. Chemical or structural modifications to crRNAs, such as introducing Locked Nucleic Acids (LNAs) or optimizing the spacer sequence, may also increase specificity and prevent off-target activation.

Simultaneously, innovative delivery methods, such as tissue-specific AAV serotypes, are being developed to improve the targeting of infected tissues both in vivo and in vitro. These approaches seek to address current challenges associated with systemic delivery by introducing tissue-specific promoters or tagging with cell specific peptides for direct and precise expression of Cas13. In the case of using LNPs, engineering organ- or tissue-specific LNPs by modifying lipid composition or adding targeting ligands (e.g., transferrin for brain targeting) represents a promising direction for exploration. Further efforts in bioengineering smaller Cas13 variants or modifying non-essential Cas13 regions while preserving RNA cleavage activity are also necessary to overcome the size limitations associated with AAV delivery. Modifications to both AAV vectors and Cas13 proteins may additionally help to reduce immunogenicity, improving the safety and efficacy of future therapeutic applications.

Furthermore, Cas13 holds significant potential as a versatile antiviral platform. By targeting conserved RNA sequences common to various RNA viruses, it could be engineered to combat multiple viral threats with minimal modifications, offering a versatile solution to emerging infectious diseases.

Each subtype of the Cas13 family exhibits unique properties in terms of protein size, crRNA requirements, and targeting efficiency and cleavage activity against different viruses. Different members of the Cas13 family are used to combat viruses in different cell lines. Numerous factors, including Cas13 orthologs, target regions within viral genomes, crRNA design, and levels of Cas13 protein expression levels, can contribute to the variability in antiviral efficacy and inhibitory effects on different viruses across different hosts. Therefore, optimizing these elements is essential to enhance the antiviral efficacy of Cas13.

Combining Cas13 with classical ARTs represents a promising future strategy for combating RNA viral infections. While ARTs inhibit key viral enzymes and replication steps, Cas13 directly targets and degrades viral RNA in a sequence-specific manner, offering a complementary mechanism of action. This combinatorial approach could enhance antiviral efficacy by simultaneously attacking multiple stages of the viral life cycle and reducing the likelihood of drug resistance. Moreover, by enabling dose reductions of traditional drugs, such a strategy may lower systemic toxicity and improve patient tolerability. Importantly, targeting both viral and host components through different modalities may provide robust, multitargeted suppression of infection and viral rebound.

### 5.2. Ethical Considerations

Cas13-based therapies, though promising, face several challenges that must be addressed to ensure in vivo safety, efficacy, and accessibility. A key concern is the potential for off-target effects, as Cas13’s RNA-targeting mechanism, while transient, could inadvertently affect the host transcriptome. Rigorous preclinical testing is crucial to mitigate these risks and ensure the safety of such therapies. Another critical issue is the immunogenicity and long-term safety of Cas13 proteins and their delivery systems. The risk of immune reactions must be thoroughly evaluated, prompting the exploration of immune-evasive Cas13 variants or alternative non-viral delivery strategies that minimize immune recognition. Additionally, as these advanced therapies progress, ensuring equitable global access is imperative. Populations in low- and middle-income countries, which are often most affected by RNA viral outbreaks, must benefit from these innovations. Global collaboration will play a vital role in making these potentially life-saving technologies accessible to all, regardless of socioeconomic barriers. For readers interested in learning more about ethical issues related to CRISPR/Cas technologies, several comprehensive and dedicated reviews have been published previously [72,73].

## 6. Conclusions

The Cas13 system represents a breakthrough in antiviral research, offering a novel approach to targeting RNA viruses. Its unique ability to degrade viral RNA with high specificity provides a promising alternative to traditional antiviral therapies. Cas13’s programmability and broad-spectrum potential make it a versatile tool in the fight against RNA viruses, especially those that mutate rapidly, such as SARS-CoV-2 and HIV. However, challenges related to off-target effects, delivery, and immunogenicity must be addressed before Cas13 can be widely adopted in clinical practice. With ongoing research and development, Cas13 has the potential to revolutionize antiviral therapies and provide new solutions for combating some of the world’s most pressing viral threats.

## Figures and Tables

**Table 1:** Comparison of the mechanisms of action, advantages, and disadvantages of Cas9, Cas12, and Cas13.

CRISPR /Cas System	Mechanism of Action	Strengths	Weaknesses
Cas9	Cas9 contains many domains, among which the HNH and RuvC domains are particularly important. When the crRNA synthesized by crRNA and tracrRNA binds to Cas9, the other domains will work together to assist crRNA in identifying the PAM site in the target gene. After locating the target gene, the HNH and RuvC domains will simultaneously cut the double-stranded strands of the target gene.	crRNA is synthesized from crRNA and tracrRNA, which is more accurate for locating the PAM site of the target gene.	1. Cas9 has no trans-cleavage activity, and in vitro detection is difficult. 2. Cas protein can cut dsDNA, but the range is relatively limited.
Cas12	The Cas12 system does not require tracrRNA and RNaseIII to process crRNA, but the nuclease in the WED domain of the Cas12 protein directly catalyzes the synthesis of crRNA. The processed crRNA directly binds to the Cas12 protein to locate the target gene and is sheared by the RuvC domain.	1. The Cas12 system only needs crRNA to bind to the Cas12 protein for detection, and the test is easy. 2. The Cas12 protein has trans-cleavage activity and can be independently detected in vitro.	1. The PAM site sequence of the Cas12 system is TTTV (V is A, G, or C), and crRNA design is difficult. 2. The Cas12 protein can only cut dsDNA and ssDNA and cannot recognize and detect RNA.
Cas13	The Cas13 system does not require tracrRNA. The pre-crRNA is processed into mature crRNA by the HEPN-2 domain, which then recognizes the target gene after binding to the crRNA and is cut by the HEPN domain in the Cas13 protein.	1. The Cas13 system has a PFS sequence that is equivalent to the PAM sequence. This sequence is composed of A, U, or C at the 3′ end of the spacer sequence, which increases the fault tolerance of the Cas13 protein. 2. The Cas13 protein can tolerate mismatches between crRNA and bases outside the seed region of the target sequence, which has almost no effect on the cutting efficiency. 3. The Cas13 protein has trans-cutting activity and can be tested in vitro alone.	Cas13 protein can only mechanically cut RNA. If DNA detection is required, DNA must be converted to RNA in vitro for detection, which increases the risk of nucleic acid contamination during the experiment.

**Table 2:** Comparison of the features of the Cas13 members.

Subtype	VI-A	VI-B	VI-C	VI-D	Cas13X	hfCas13X	Cas13Y
Cas effector	Cas13a	Cas13b	Cas13c	Cas13d (CasRx)	Cas13X	hfCas13X	Cas13Y
Accessory proteins	Cas1 and Cas2	Csx27/28	Unknown	Cas1 and Cas2	None	None	None
Homologs/complexes	*Leptotrichia* spp. *Lahnospiraceae bacterium*	*Bergeyella zoohelcum*, *Prevotella buccae*	*Fusobacterium perfoetens*	*Leptotrichia shahii*	Metagenomic source	Engineered Cas13X	Metagenomic source
Size	~1250 aa	~1150 aa	~1120 aa	~930 aa	~775 aa	~775 aa	~775 aa
Architecture	REC and NUC lobe	Pyramidal	Uncharacterized	REC and NUC lobe	Compact single-lobe	Compact single-lobe	Compact single-lobe
Pre-crRNA processing site	Helical-1 and HEPN-2	RRI-2 (Lid)	Unknown	HEPN-2	HEPN domain	HEPN domain	HEPN domain
Pre-crRNA mechanism	Acid–base	Acid–base	Unknown	Acid–base	Not reported	Not reported	Not reported
ssRNA cleavage preferences	U- and A-rich	Pyrimidine (U)	Unknown	U	U and others	Reduced off-target	Likely U-rich
Protospacer-flanking sequence	5′ non-G	5′ non-C, 3′ NAN	No restriction	No restriction	Unknown	Unknown	Unknown
Small accessory proteins	None	Csx27/28	WYL-domain	None	None	None	None
Orientation (repeat→spacer)	5′→3′	3′→5′	Unknown	5′→3′	5′→3′	5′→3′	5′→3′
Repeat length (mature)	27–32 nt	36–88 nt	Unknown	30 nt	~28–30 nt	~28–30 nt	~28–30 nt
Repeat architecture	Stem-loop	Distorted stem-loop	Unknown	Stem-loop	Simple stem-loop	Simple stem-loop	Simple stem-loop
Recognition mechanism	Sequence + structure	Structure	Unknown	Sequence + structure	Likely structural	Enhanced fidelity	Likely structural
Spacer mismatch-sensitive	Seed region, HEPN switch	Central	Unknown	Internal, 3′	Limited data	High specificity	Limited data
Reference	Abudayyeh et al. [23]	Smargon et al. [27]	–	Konermann et al. [26]	Xu et al., 2021 [29]	Xu et al., 2021 [29]	Xu et al., 2021 [29]

**Table 3:** Overview of Cas13 therapeutic strategies and efficacy against RNA viruses.

Virus Type	Publication	Strategy	Efficacy
SARS-CoV-2	[32]	Cas13d with DNA constructs co-transfected into lung epithelial cells (A549).	GFP fluorescence reduced by 86%, mRNA expression by 83%.
	[33]	Cas13d in Vero E6 cells targeting Alpha, Beta, and Omicron variants using lentivirus delivery.	Viral titer inhibited by ~95% at 24 hpi, up to 97% with combined crRNAs.
	[34]	Cas13a mRNA and crRNA delivered to hamsters via nebulizer; tested in vitro and in vivo.	72% plaque reduction in vitro; 57% lung viral RNA reduction and abrogated weight loss in hamsters.
	[35]	Cas13a targeting spike protein in HepG2 and AT2 cells via lentivirus delivery.	Silencing efficiency >99.9% by qPCR.
	[36]	Cas13b targeting pseudoknot in hACE2 transgenic mice and in vitro.	Reduced viral replication by 99%; spike protein expression significantly lowered.
	[37]	Cas13d with 29 crRNAs targeting conserved regions of SARS-CoV-2 genome; tested in replicon and reporter assays.	Efficient suppression of SARS-CoV-2 replicon; crRNAs also inhibited SARS-CoV, showing broad antiviral potential.
	[38]	Cas13d with 50 crRNAs targeting conserved regions of ORF1ab region (NSP13 and NSP14) of SARS-CoV-2 genome; tested coronaviral nucleocapsid protein (NP) levels by Western blotting.	Achieved >99% silencing efficiency on nucleocapsid transcripts in human cells which are infected with coronavirus 2.
	[36]	Cas13b with 12 crRNAs targeting the pseudoknot site upstream of ORF1b; tested in replicon and infectivity in Vero E6 and hACE2 transgenic mice.	Reduced expression of the spike protein and attenuated viral replication by 99%.
Human Enterovirus	[39]	AAV-delivered Cas13 system targeting conserved viral RNA sequences designed via bioinformatics pipeline.	Reduced viral titers by >99.99% in vitro; prophylactic and therapeutic inhibition in mice prevented death in lethal challenge.
Dengue	[40]	Cas13a/crRNA complex transfected into DENV-2-infected Vero cells.	RNA copy inhibition ~95% and plaque reduction ~84% at day 3.
	[41]	Cas13b RNP delivered via virus-like particles (VLP) to human primary cells.	Efficient suppression of dengue virus infection.
	[42]	LNP-formulated mRNA-encoded Cas13a and crRNA applied in DENV infected mice.	Improved the survival of all infected animals and significantly decreased serum viral titers, with no collateral cleavage observed.
Influenza A (IAV)	[34]	Cas13a mRNA and crRNA were tested in mouse model.	94% mRNA expression reduction and significant protection in treated animals.
	[43]	Cas13a in chicken fibroblast cells targeting IAV strains WSN and PR8.	Two- to fourfold reduction in infection by plaque assays.
	[44]	mRNA-encoded LbuCas13a, along with two crRNAs targeting H1N1 and H3N2 strains, was tested in A549 cells and hamsters.	RNA degradation was observed when delivered 24 h post-infection in vitro, along with a 1–2 log reduction in viral titers in hamsters
HIV	[45]	Cas13a targets HIV in HEK293T cells.	viral replication of HIV was suppressed and the RNA levels were reduced.
	[46]	RfxCas13d with HIV-specific crRNAs targeting in primary CD4^+^ T cells and reactivated latent HIV.	Inhibited HIV-1 replication.
PRRSV	[47]	Cas13b targeting ORF5 and ORF7 in cell culture.	Gene knockdown and significant GFP fluorescence reduction.
Hepatitis C Virus	[48]	Cas13a targeting internal ribosomal entry site (IRES) in Huh7.5 cells.	Demonstrated 85% inhibition of luciferase activity.
Phage	[49]	Versatile Cas13a for phage genome editing.	Effective phage targeting and genome editing.
Borna	[50]	pspCas13b was used in BoDV-1 infected 293T cells.	Suppressed BoDV-1 in both acute and persistent infections.

## Data Availability

No new data were created or analyzed in this study. Data sharing is not applicable to this article.

## Abbreviations

The following abbreviations are used in this manuscript:

UTR	Untranslated Region
ORFs	Open Reading Frames
RdRPs	RNA-Dependent RNA Polymerases
PRRSV	Porcine Reproductive and Respiratory Syndrome Virus
AIVs	Avian Influenza Viruses
ART	Antiretroviral Therapy
CRISPR	Clustered Regularly Interspaced Short Palindromic Repeats
Cas	CRISPR/Associated Proteins
HEPN	Higher Eukaryotes and Prokaryotes Nucleotide-Binding
WHO	World Health Organization
HIV	Human Immunodeficiency Virus
HAART	Highly Active Antiretroviral Therapy
NRTI	Nucleoside Reverse Transcriptase Inhibitor
NNRTI	Non-nucleoside Reverse Transcriptase Inhibitor
INSTI	Integrase Strand Transfer Inhibitor
PI	Protease Inhibitor
LNPs	Lipid Nanoparticles
DENV	Dengue Virus
crRNA	CRISPR RNA
CPE	Cytopathic Effect
IAV	Influenza A Virus
ssRNA	Single-Stranded RNA
HCV	Hepatitis C Virus
IRES	Internal Ribosome Entry Site
HDV	Hepatitis Delta Virus
BoDV-1	Borna Disease Virus
AAVs	Adeno-Associated Viruses
LNAs	Locked Nucleic Acids
FDA	Food and Drug Administration
EMA	European Medicines Agency
ICH	International Council for Harmonisation of Technical Requirements for Pharmaceuticals for Human Use
CDRH	Center for Devices and Radiological Health
EUA	Emergency Use Authorization
nAMD	Neovascular Age-related Macular Degeneration
CNV	Choroidal Neovascularization
aa	Amino acid
nt	Nucleotide
